# Reaction-time task reliability is more accurately computed with permutation-based split-half correlations than with Cronbach’s alpha

**DOI:** 10.3758/s13423-024-02597-y

**Published:** 2024-10-23

**Authors:** Sercan Kahveci, Arne C. Bathke, Jens Blechert

**Affiliations:** 1 Department of Psychology, Paris-Lodron-University of Salzburg, Hellbrunner Straße 34, 5020 Salzburg, Austria; 2 Centre for Cognitive Neuroscience, Paris-Lodron-University of Salzburg, Hellbrunner Straße 34, 5020 Salzburg, Austria; 3 Intelligent Data Analytics Lab, Department of Artificial Intelligence and Human Interfaces, Paris-Lodron-University of Salzburg, Hellbrunner Straße 34, 5020 Salzburg, Austria

**Keywords:** Reliability, Cognitive tasks, Reaction time analysis, Psychometrics/testing

## Abstract

While it has become standard practice to report the reliability of self-report scales, it remains uncommon to do the same for experimental paradigms. To facilitate this practice, we review old and new ways to compute reliability in reaction-time tasks, and we compare their accuracy using a simulation study. Highly inaccurate and negatively biased reliability estimates are obtained through the common practice of averaging sets of trials and submitting them to Cronbach’s alpha. Much more accurate reliability estimates are obtained using split-half reliability methods, especially by computing many random split-half correlations and aggregating them in a metric known as permutation-based split-half reliability. Through reanalysis of existing data and comparison of reliability values reported in the literature, we confirm that Cronbach’s alpha also tends to be lower than split-half reliability in real data. We further establish a set of practices to maximize the accuracy of the permutation-based split-half reliability coefficient through simulations. We find that its accuracy is improved by ensuring each split-half dataset contains an approximately equal number of trials for each stimulus, by correcting the averaged correlation for test length using a modified variant of the Spearman–Brown formula, and by computing a sufficient number of split-half correlations: around 5,400 are needed to obtain a stable estimate for median-based double-difference scores computed from 30 participants and 256 trials. To conclude, we review the available software for computing this coefficient.

## Introduction

Within the past 2 decades, the field of experimental psychology has seen a surge in indirect measures such as the implicit association task, the dot-probe task, and the approach–avoidance task (AAT). Theories behind these indirect measures claim that they tap into attitudes, associations, as well as behavioral and attentional biases outside the participants’ awareness, thus evading the influence of deliberate processes such as social desirability and experimenter demand (De Houwer, 2006). The approach–avoidance task, for example, has provided evidence that individuals are faster to avoid than approach the stimuli associated with their fears and anxieties (social anxiety: Heuer et al., 2007; contamination fear: Najmi et al., 2010; spider fear: Rinck & Becker, 2007), and they are faster to approach than avoid the stimuli associated with substances they frequently consume or desire to consume (cannabis: Cousijn et al., 2011; food: Kahveci et al., 2020a; alcohol: Kersbergen et al., 2015). With their focus on speed and accuracy, the aforementioned biases are thought to occur in the background of the cognitively demanding task at hand, and thus tap into partially implicit response tendencies (De Houwer et al., 2009). To quantify these potentially implicit attitudes, associations, and biases, researchers aggregate the participants’ reaction times (RTs) into a bias score by contrasting mean RTs and error rates in response to one category of trials, such as spider stimuli, versus another, often neutral category, such as leaf stimuli (Rinck et al., 2021).

Methodological standards for indirect psychological tasks lag much behind those of self-report scales: Psychologists have come to mistrust self-report scales with reliabilities below 0.8, but the results of experimental tasks with reliabilities hovering around zero, such as the classical dot-probe task, are still being published (Van Bockstaele et al., 2020). This use of unreliable paradigms has undoubtedly contributed to the replication crisis in psychological science: The less reliable a score is, the more likely it is that the differences between individuals’ scores are caused by measurement error rather than by the true construct. As such, any significant correlations that emerge from an unreliable score are more likely to be based on that measurement error than on true underlying relationships (Tabachnick et al., 2019). It has therefore been suggested that the reliability of experimental paradigms should always be reported (Parsons et al., 2019), which is currently only done in a minority of studies. In our own review of the AAT literature, we found that only 38 out of 163 individual experiments reported a measure of reliability (Kahveci et al., 2023).

We intend to contribute to recent efforts (Parsons et al., 2019; Pronk et al., 2022) to make adequate reliability assessment of implicit measures feasible and accessible. Therefore, we will provide a didactic introduction to the family of single-session reliability metrics and further examine their accuracy when applied to RT data, using the approach–avoidance task as an example. In the introduction, we will lay out how RT data are structured in this task and how this differs from the self-report data that reliability metrics like Cronbach’s alpha are typically applied to, before elaborating what this implies for the practice of computing the reliability of RT tasks. To do so, we will discuss the mathematics behind Cronbach’s alpha, and what makes it inapplicable to most RT tasks. Next, we discuss the benefits and pitfalls of the various split-half reliability coefficients that are in use or have been proposed, such as the odd–even split-half, the permutation-based split-half, and the Monte Carlo split-half. In three simulation studies, we examine whether our reservations about Cronbach’s alpha and the odd–even split-half method are justified, we determine the optimal number of splits for calculating the permutation-based split-half reliability, and we determine whether the 95% confidence intervals provided by this coefficient are accurate. We follow up by verifying whether our simulated findings hold in actual data by reanalyzing six datasets and meta-analyzing several studies that reported multiple measures of reliability. We finish with a discussion of recommendations and an overview of the available software in R.

### Characteristics of reaction-time task data and illustration using the AAT

**Fig. 1:**
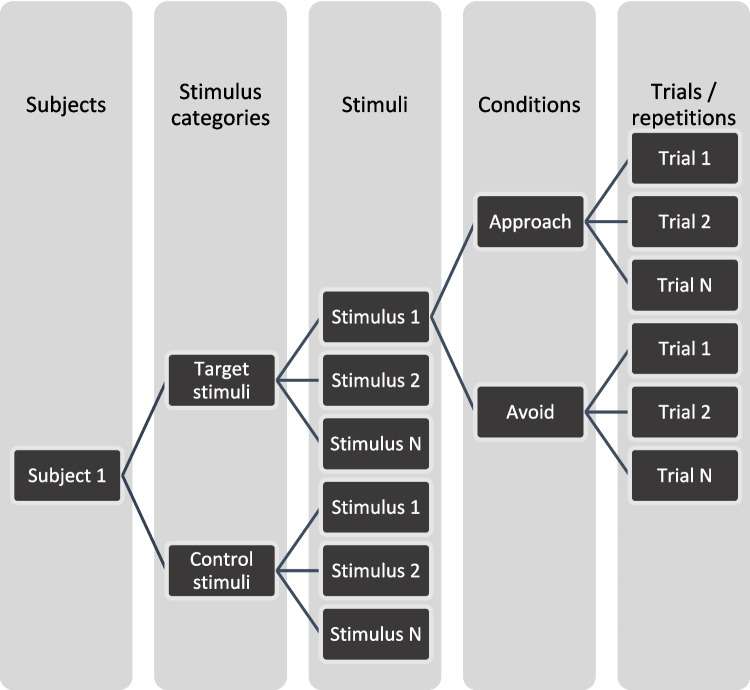
Data structure of the AAT

To discuss the data structure of typical RT tasks for implicit bias assessment, we will use the AAT as an example. The structure of this task is illustrated in Fig. 1. In the AAT, participants rapidly respond to individual stimuli by either approaching or avoiding them (typically by pulling or pushing a joystick), on the basis of a stimulus feature that they have been informed about before the block starts. For example, before Block 1, they may be instructed to approach foods and avoid objects, and before Block 2, they may be instructed to instead avoid foods and approach objects. This gives rise to 2 × 2 unique combinations of image type and response direction. Participants typically view many different stimuli per stimulus category (e.g., different species of spiders), and the same stimulus is often displayed many times.

After the data are collected, extreme RTs and incorrect responses are removed, and the mean approach RT is subtracted from the mean avoid RT for each stimulus category to gain a measure of how much faster or slower each category was approached instead of avoided. For example, a bias score of 100 for food stimuli would mean food stimuli were on average approached 100 ms faster than they were avoided. The resulting approach–avoid difference score of a target stimulus category is then subtracted from the approach–avoid difference score of a neutral stimulus category, resulting in a double difference score that is unconfounded by the ease of one movement (e.g., pulling a joystick) versus the contrasting movement (e.g., pushing a joystick, e.g., here: 20 ms).

There are three important characteristics by which the practices around RT tasks differ from those around self-report scales: individual self-report items can readily be linked to their counterpart across participants, while individual RTs typically cannot, because trials tend to be displayed in random order and repeat the same stimuli multiple times. Second, RTs are often removed when they are too extreme or associated with an erroneous response, which means that trial numbers differ across participants, whereas responses to individual self-report items are rarely removed. Third, RT tasks are often scored by subtracting one mean from another, while self-report scales are more often scored by summing or averaging responses. We argue that reliability coefficients are only appropriate for RT tasks if they take these characteristics into account. Unfortunately, this is often not the case.

### Introduction to Cronbach’s alpha and its limitations in reaction-time measures

**Fig. 2:**
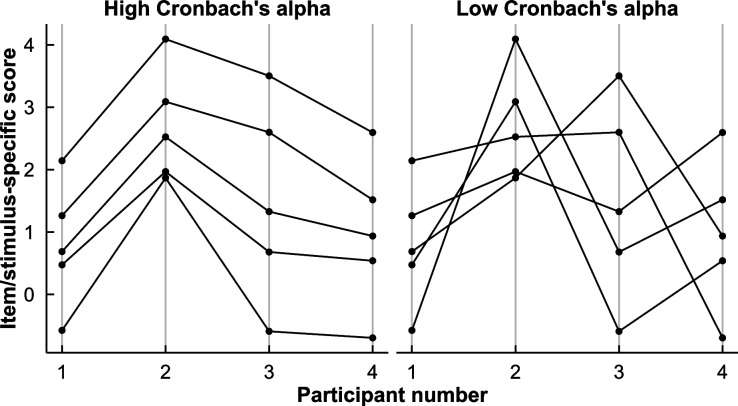
Illustration of the rationale behind Cronbach’s alpha. The *x*-axis represents individual participants, and points and lines represent individual scores of questionnaire items or individual stimuli. The points in each plot average to the same score per participant, but covariance between individual stimuli is high on the left, indicating high reliability (α = 0.96), and low on the right, indicating low reliability (α = 0.52)

Cronbach’s alpha measures reliability by quantifying to what extent the different scores of items or stimuli covary across participants. Cronbach’s alpha values are higher when scores of items or stimuli increase or decrease in synchrony across participants, as depicted in Fig. 2. A high alpha value thus indicates high reliability in the sense that variance in the individual stimulus or item scores can be attributed to a single variable measured by all items or stimuli, rather than to measurement error or idiosyncrasies specific to the individual items or stimuli. In such a situation, those with the highest scores in the sample would have the highest scores on all individual items or stimuli, while the lowest scorers would likewise have the lowest scores on all individual items and stimuli. Cronbach’s alpha is displayed in Eq. 1, where *p* represents the number of items/stimuli, and *X*_*i*_ and *X*_*j*_ represent the scores for items/stimuli *i* and *j*:

1 $$\alpha =\frac{p}{p-1}\left(1-\frac{{\sum }_{i}^{p}Var\left({X}_{i}\right)}{\sum_{i}^{p}\sum_{j}^{p}Cov\left({X}_{i},{X}_{j}\right)}\right)$$

There is often no way to directly assign a single RT to a single item slot in Cronbach’s alpha, because outlying or incorrect responses need to be excluded, and because the score under study is often a difference score while there are many ways to match individual trials to each other for subtraction. To nevertheless compute Cronbach’s alpha in an RT task, researchers therefore have resorted to various workarounds that distort the accuracy of the measure.

#### Cronbach’s alpha often leads researchers to include outliers and errors in its calculation

Cronbach’s alpha cannot be computed when datapoints are missing due to outlier or error trial removal. This limitation has led researchers to include errors and outliers in their data when computing Cronbach’s alpha (Staugaard, 2009; Waechter et al., 2014), producing reliability values that can be expected to be lower than when these trials would be excluded. The scores that are actually used in the main analyses often do not include these errors or outliers (Effting et al., 2016; Kersbergen et al., 2015; Watson et al., 2013), making it so that the reported reliability coefficient does not represent these scores.

#### Cronbach’s alpha is often computed for something other than the variable of interest

Relatedly, there are also cases in which researchers compute Cronbach’s alpha for a wholly different variable than was used in analyses: it is not commonly known how to compute Cronbach’s alpha for an RT difference score involving two sets of stimuli, so some researchers instead compute it for the mean RT of each condition, while the actual variable used in the study is a difference score (e.g., Manchery et al., 2017; Sklenarik et al., 2024; Struijs et al., 2017). This is not only uninformative but also misleading, since mean RTs are almost always highly reliable (> 0.90), while difference scores may not be.

#### Cronbach’s alpha does not capture all variance in the data when applied to aggregated reaction-time subscores

**Fig. 3:**
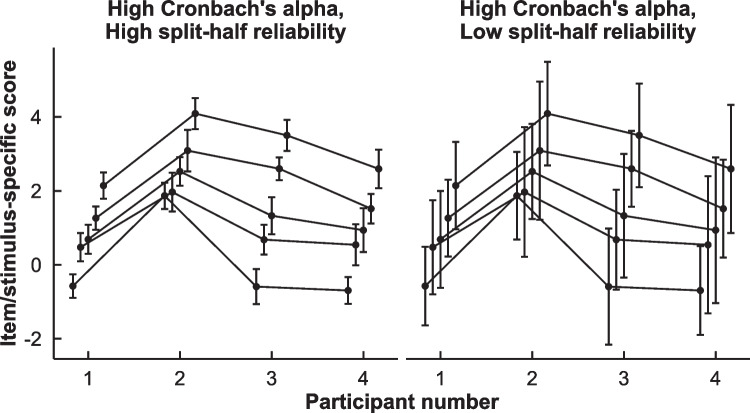
Illustration of the inaccuracy of Cronbach’s alpha when applied to trial-level data through averaging. Each point and confidence interval is based on the mean and standard error of RTs of simulated responses to six repetitions of the same image. Cronbach’s alpha is 0.96 for both plots, but when reliability is computed with permutation-based split-halves, which are influenced by individual RT variance, the data on the left are reliable, *r* = 0.94, and those on the right are unreliable, *r* = 0.45

Cronbach’s alpha is often applied to RT tasks by computing averages or bias scores for each stimulus within each participant and submitting the resulting stimulus bias scores to Cronbach’s alpha. Thus, each stimulus-specific subscore is treated as one would treat a self-report item on a scale. This approach predominates in the AAT literature (Brom et al., 2014; Cousijn et al., 2011, 2013, 2014, 2015; Klein et al., 2011; Kong et al., 2015; Lobbestael et al., 2016; Reinecke et al., 2010; Watson et al., 2013) but has also been used elsewhere (Bohne et al., 2021). Other studies aggregate series of trials into subparcels in a similar manner but on the basis of position within a block rather than the stimulus (Atwood & Axt, 2021). We identify three issues with this approach. First, it unduly grants some trials more influence than others when subscores were based on unequal numbers of trials, since Cronbach’s alpha represents the reliability of the mean of all subscores and thus assigns equal weight to subscores that were based on one RT versus five RTs. Second, since RTs are aggregated before being submitted to Cronbach’s alpha, the error of the subscores is not directly included within the computation of the reliability. A subscore-based Cronbach’s alpha is thus only sensitive to the concordance between subscores, not to other indicators of (un)reliability such as trial-level RT variance. This is illustrated in Fig. 3, where trial-level variance is displayed as confidence intervals surrounding subscores; the confidence intervals are wide on the left and narrow on the right, implying inaccurate measurement on the left and more precise measurement on the right. Yet Cronbach’s alpha gives the same reliability for both datasets, as that level of variance is removed before computing the coefficient. A third concern, discussed earlier by Parsons et al. (2019), is that Cronbach’s alpha assumes that the participants would respond similarly to the same stimulus or subparcel compared to another stimulus or subparcel; a departure from this pattern is assumed to represent a departure from reliability. However, this is generally not assumed by RT researchers and thus represents a mismatch between the method and the theoretical model of the researchers using it.

#### Cronbach’s alpha systematically underestimates reliability

The final issue of Cronbach’s alpha is applicable not just to RT tasks but is relevant to the full literature: Cronbach’s alpha is a lower bound to reliability in general, and thus systematically underestimates it (Sijtsma, 2009). Less negatively deviated alternatives have been proposed, such as McDonald’s omega, but these suffer from the same issues listed in previous paragraphs.

Taken together, it can be concluded that Cronbach’s alpha is only applicable to RT data under very specific circumstances which rarely apply in practice (i.e., no trial exclusions); and even then, it will underestimate reliability. Given these issues, it is troubling that this reliability coefficient is very common in the literature on implicit bias—for example, in the AAT (e.g., Brom et al., 2014; Lobbestael et al., 2016; Sklenarik et al., 2024; Struijs et al., 2017; Watson et al., 2013), the dot-probe task (e.g., Ataya et al., 2012; Christiansen et al., 2015; Jones et al., 2018; Manchery et al., 2017; Szasz et al., 2012; Tian & Smith, 2011; Van Duijvenbode et al., 2017; van Ens et al., 2019), the Stroop task (e.g., Spanakis et al., 2019), and the implicit association task (e.g., Atwood & Axt, 2021; Cummins et al., 2022; Tabatabaei & Beldona, 2024).

### Reliability assessment at the trial level through variants of split-half reliability

To know how well the scores on two repeated measurements of an RT task will correlate, the split-half reliability coefficient is preferable, since this method can handle variability in the number of trials, including when outlying and error trials are excluded. In the following section, we will expand on the review of Pronk et al. (2022) on the available split-half methods for implicit measures. We follow up with four studies in which we apply these metrics and Cronbach's alpha to simulated and real data in the empirical section of this paper, with the goal of estimating and improving the accuracy of the reliability metrics.

#### Single split-half reliability

To compute a *single split-half reliability* coefficient, one divides each participant’s data into two equally sized halves, computes scores per participant from each half, and then correlates the participants’ scores in one half with their equivalent scores in the other half. Since this split-half correlation is computed from scores based on only half of all trials, it is lower than would be the case if the full task were administered twice, since tests with more items or trials tend to be more reliable. The Spearman–Brown formula (Eq. 2) is used to estimate what the hypothetical correlation (*r*_*kk*_) between these split-halves would be if their number of trials or items were multiplied by a factor *k,* given the original reliability (*r*_11_). Therefore, the formula (with *k* = 2) is applied to the raw split-half correlation to obtain the reliability for the full experiment:

2 $${r}_{kk}=\frac{k\cdot {r}_{11}}{1+(k-1)\cdot {r}_{11}}$$

In the RT task literature, it is common for the data to be split by even/odd trial numbers (*odd–even split-half*), or by the middle of the experiment (*first–second split-half*). This measure produces different results depending on the way the data is split, and as such, the outcome of an individual split is arbitrary and likely to be unrepresentative of how the task might correlate with its own replication.

#### Permutation-based split-half reliability

The arbitrariness of any single split can be overcome by computing the split-half correlation of many random splits and averaging the result to produce the *permutation-based split-half reliability* (used by, e.g., Chapman et al., 2019; Kahveci et al., 2020a; Parsons et al., 2019; Waechter et al., 2014), also known as the randomized or bootstrapped split-half reliability. Although this method is more resource intensive, it is likely to be a more accurate estimate of reliability than either the single split-half or Cronbach’s alpha, since it averages out the random fluctuations that can originate from arbitrarily splitting the data into two halves or multiple subscores. Before the permutation-based split-half can be used, three important issues need to be tackled: stratification, correlation aggregation, and the handling of negative values in the Spearman–Brown formula.

##### How to split the data?

The goal of a split-half reliability coefficient is to approximate the test–retest reliability of a true replication of the experiment, which would contain the same participants, the same conditions, the same stimuli, and the same number of trials in each; the random splits have to include equal numbers of trials of each condition within each participant, and if possible, each stimulus (Pronk et al., 2022). After all, if the data is split such that participant A has different stimuli in each half, then the halves are not each other’s direct replications, and the resulting split-half reliability coefficient would not be valid. Instead of randomly dividing the trials of all individuals into two halves, we therefore ensured in the present studies that each half featured an approximately equal number of trials for each condition. Furthermore, we explored the merits of more thorough stimulus-level stratification by employing a splitting algorithm wherein (a) trials within each stratum (e.g., stimulus within condition within participant) are divided in pairs of two, leaving 0 or 1 leftover trial per stratum; (b) all pairs are divided in two, for each trial to be assigned to a half; (c) all leftover trials are pooled on a higher stratum (e.g., condition within participant) to be assigned randomly to one or the other half. This algorithm allows for stratified splitting of small strata while preserving near-equal sample sizes between both resulting halves.

##### How to aggregate the individual correlations?

Previous research into permutation-based split-half reliability has relied on and recommended computing the simple mean of the permutated split-half correlation coefficients (Parsons et al., 2019; Pronk et al., 2022), but it is known from previous research (Shieh, 2010) that simple means of correlations are biased towards zero. The Fisher *z* transformation is commonly used to counteract this bias when averaging correlations, but it is unfortunately biased in the opposite direction, yielding average correlations too close to 1 or − 1 (Silver & Dunlap, 1987). It was found (Shieh, 2010) that the correlation aggregation methods of Olkin and Pratt (1958) reduced this bias, which should slightly improve the accuracy of the permutation-based split-half reliability coefficient. Instead of simply averaging raw correlations, we therefore used their methods in the present studies.

##### How to apply the Spearman–Brown formula?

A common issue when using the Spearman–Brown formula is that applying it to negative correlations produces extreme and often impossible values (e.g., below − 1). In the present studies, we therefore investigate whether more accurate results are obtained by replacing negative values with zero or by modifying the formula such that the same degree of change is applied to negative correlations as is done to positive correlations. Another issue we will address in the context of permutation-based split-half reliability is whether more accurate results are obtained when applying the Spearman–Brown formula to the raw correlations or to their average.

#### Monte Carlo split-half

The Monte Carlo split-half (Williams & Kaufmann, 2012) was recently described as a more robust way of computing reliability with nonlinear scoring algorithms (Pronk et al., 2022). To compute this coefficient, two datasets are sampled from the original dataset *with replacement*, such that the resulting datasets are as large as the original. After this, scores for each participant are computed from both datasets and correlated. Like with the permutation-based split-half, this is repeated many times and all correlations are averaged to compute the reliability coefficient, but without the need for any subsequent application of the Spearman–Brown formula to correct for the use of smaller samples. This method runs the risk of heavily overestimating reliability, as individual trials can end up in both datasets (due to sampling with replacement), causing an artificial similarity between the two sets. We will examine whether our concerns about this method are justified in our first study.

### Interim summary and the present study series

In our introduction of reliability estimation in RT-based implicit measure studies, we discussed the characteristics and pitfalls of Cronbach’s alpha and several split-half reliability coefficients. Cronbach’s alpha, we discussed, cannot readily be applied to RT data in most occasions, and will likely produce inaccurate reliabilities; meanwhile, single split-half reliability coefficients are subject to random error. Permutation-based split-half reliability may be more accurate, especially when stratifying the splits to ensure the split-halves represent direct replications of each other. Lastly, the Monte Carlo split-half is likely to overestimate reliability due to the inclusion of a large amount of identical data in both split halves.

In Study 1, we examined whether our reservations regarding the aforementioned methods were justified by analyzing to what extent each of the reliability coefficients predicted the correlation between two simulated measurements of the same variable, using only the reliability of the individual measurements. Since the permutation-based split-half reliability coefficient can be computationally intensive, Study 2 examined the minimum number of splits needed to accurately compute it. In Study 3 we evaluated the accuracy of the 95% confidence intervals generated with the permutation-based split-half reliability method. Lastly, in Study 4, we compared whether real data reported in the literature also showed the differences between Cronbach’s alpha and split-half reliability that we found for simulated data in Study 1.

## Study 1: Comparing five reliability coefficients

### Introduction and methods

To compare the reliability coefficients, we generated datasets and analyzed to what extent each reliability coefficient enabled us to predict the correlation between two measurements of the same variable in the same participant (test–retest correlation) using only the reliability of the individual datasets (within-session reliability) as predictors. By using simulated data, we were able to manipulate various aspects of the data and analyze in what ways these aspects distort the accuracy of the reliability estimate.

To examine the accuracy of reliability coefficients when applied to double-difference scores, we implemented a data simulation procedure to generate data with specific Cronbach’s alpha values for stimulus-specific bias scores within stimulus category (e.g., target stimulus category or control stimulus category), correlations between bias scores of the separate stimulus categories, and *SD* of the RTs to individual stimuli (see Appendix A for details). We generated two datasets for each permutation of within-stimulus-category bias score reliabilities of 0, 0.2, 0.4, 0.6, 0.8, and 1; within-stimulus-category bias score variances of 2, 4, 6, 8, and 10; within-stimulus RT *SD* of 2, 4, 6, 8, and 10; and intercategory correlations of − 0.5, 0, and 0.5. Each of these 2 × 6 × 5 × 5 × 3 = 900 datasets had 40 participants and eight stimuli per category, with eight approach and eight avoidance trials per stimulus, thus giving 64 approach and 64 avoid trials for each of the two stimulus categories within participant, totaling 256 trials.

We also examined the accuracy of reliability coefficients when applied to single-difference scores and simple averages. For single-difference scores, we generated six datasets instead of two, for each permutation of the aforementioned values for within-category reliability, within-category variance, and within-stimulus RT *SD*; each dataset had eight stimuli within only one stimulus category instead of two, thus totaling 128 trials. For simple averages, we generated the same number of datasets within each aforementioned permutation, but we generated only eight trials per stimulus that were not nested within an approach or avoidance response condition, totaling 64 trials per participant.

These datasets were all split in two, creating two half-datasets with the same participants, stimuli, and responses. The “true” scores of these half-datasets originate from the same individuals and are thus perfectly correlated; for scores computed from the actual data, any deviation from perfect correlation can be attributed to measurement error and thus imperfect reliability. For both half-datasets, we computed five reliability coefficients: Cronbach’s alpha, odd–even split-half reliability, permutation-based split-half reliability, stratified permutation-based split-half reliability, and Monte Carlo split-half reliability. To compute the permutation-based and Monte Carlo split-half reliabilities, 10,000 individual random split-half reliabilities were aggregated using Eq. 2.7 from Olkin and Pratt (1958), reproduced here in Eq. 3. In the stratified permutation-based split-half, the splitting was stratified by stimulus such that each stimulus featured a near-equal number of trials in both halves. The permutation-based reliability coefficients were computed using R package *rapidsplithalf* (Kahveci, 2024).

3 $$\overline{{r }_{OP5}}=\frac{1}{n}\sum_{i}\left\{{r}_{i}\left[1+\sum_{k=1}^{5}\frac{{\left(\Gamma \left(\frac{1}{2}+k\right)\right)}^{2}\Gamma \left(\frac{N-2}{2}\right)}{{\left(\Gamma \left(\frac{1}{2}\right)\right)}^{2}\Gamma \left(\frac{N-2}{2}+k\right)}\cdot \frac{{\left(1-{r}^{2}\right)}^{k}}{k!}\right]\right\}$$

Since Cronbach’s alpha is inapplicable when the outcome measure consists of the score of one stimulus set minus that of another, we applied Eq. 4 (Lord, 1963) to nevertheless obtain the alpha-based reliability of such a difference score (*r*_*a-b*_), using the (Cronbach’s alpha) reliabilities of the two to-be-subtracted scores (*r*_*a*_ and *r*_*b*_), their *SD*s (*s*_*a*_ and *s*_*b*_), and the correlation between the two (*r*_*ab*_):

4 $${r}_{a-b}=\frac{{r}_{a}{s}_{a}^{2}+{r}_{b}{s}_{b}^{2}-2{r}_{ab}{s}_{a}{s}_{b}}{{s}_{a}^{2}+{s}_{b}^{2}-2{r}_{ab}{s}_{a}{s}_{b}}$$

We evaluated the accuracy of the different reliability coefficients based on the idea that a good single-session reliability estimate should accurately forecast a task’s test–retest correlation. We therefore computed the *observed* test–retest correlation by computing participant-specific scores in each half-dataset and correlating scores of both half-datasets with each other. We computed the ‘*predicted’* test–retest correlation based on the reliability of both individual sets using Eq. 5. This equation was adapted from Spearman (1904) to allow negative values, such that a negative predicted test–retest correlation would result if both of the single-session reliability coefficients were negative (as determined with the *sgn* function). If one single-session reliability estimate was positive while the other was negative, the sign of the predicted test–retest correlation was deemed undefined and excluded from the analysis:

5 $${r}_{predicted}=\left\{\begin{array}{cc}\sqrt{\left|{r}_{1}\right|\times \left|{r}_{2}\right|}\times \text{min}\left(\text{sgn}\ {r}_{1},\text{sgn}\ {r}_{2}\right),& \text{if}\ \text{sgn}\ {r}_{1}=\text{sgn}\ {r}_{2}\\ \text{undefined}, & \text{otherwise}\end{array}\right.$$

We evaluated the accuracy of the predicted test–retest correlations by computing their mean deviation (average error in a certain direction) and the root mean square error in comparison to their respective observed test–retest correlations.

Before we were able to compare the different reliability coefficients with each other, we had to determine how to deal with negative reliabilities submitted to the Spearman–Brown formula. Three approaches were compared: no correction, replacing negative values with zero (*nullification*), and using a modified formula (Eq. 6) that behaves akin to submitting absolute values to the formula and restoring the negative sign afterwards (*mirroring*). In the permutation-based split-half reliability coefficients, which aggregate multiple split-half correlations together, we additionally evaluated the difference between applying these corrections and the Spearman–Brown formula to the reliabilities before or after aggregating them:

6 $${r}_{kkMirrorred}=\frac{k\cdot {r}_{11}}{1+(k-1)\cdot \left|{r}_{11}\right|}$$

### Results and discussion

All reliabilities analyzed here were computed from scores based on means (e.g., mean RTs, mean difference scores). The same reliabilities were computed from scores based on medians; these followed the exact same pattern as those for means, and will not be discussed.

#### Methods of treating negative reliabilities

**Fig. 4:**
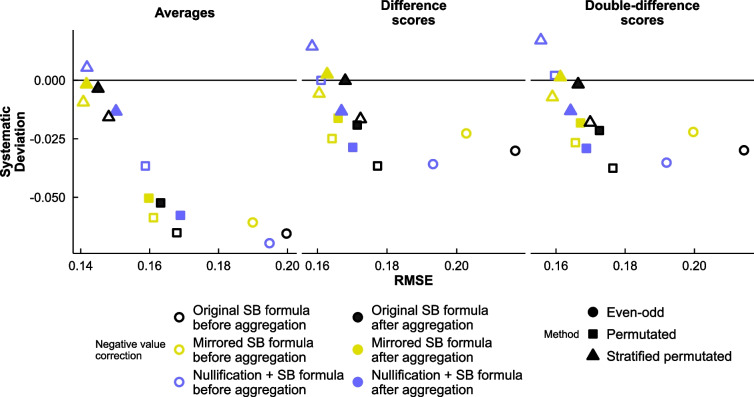
Accuracy comparison of different methods of dealing with negative split-half correlations and different sequences of aggregating split-half correlations and applying the Spearman–Brown formula. RMSE = root mean square error; systematic deviation = the mean deviation of the predicted value from the observed value, with negative values implying a systematic tendency to underestimate; SB formula = Spearman–Brown formula; mirrored SB formula = applying the same amount of change to negative correlations as to positive correlations, using Eq. 6 instead of the traditional formula; nullification = replacing negative correlations with zero before submitting them to the Spearman–Brown formula

In Fig. 4 we display the results of our exploration of how to handle negative reliabilities and whether to apply the Spearman–Brown formula before or after aggregating correlations. Spearman–Brown-corrected reliabilities with no correction of negative values were consistently less accurate than those involving any mirroring or nullification. Nullification after aggregation was also consistently less accurate and more biased than nullification before aggregation. For odd–even split-half reliability, the least deviated method was mirroring. For permutation-based split-half reliability, nullification before aggregation was less deviated and more accurate than mirroring. For stratified permutation-based split-half reliability, mirroring after aggregation was least deviated for averages and difference scores, while all methods were close in terms of error. While the point-estimates of nullification before aggregation were overall among the best in accuracy, their confidence intervals predictably excluded too many negative observed correlations, making the CIs unusable; we therefore opted to use mirroring after aggregation in the remainder of the manuscript (i.e., we aggregate the raw permutation-based split-half correlations together, and apply the modified Spearman–Brown formula from Eq. 6).

#### Accuracies of the different reliability estimators

**Fig. 5:**
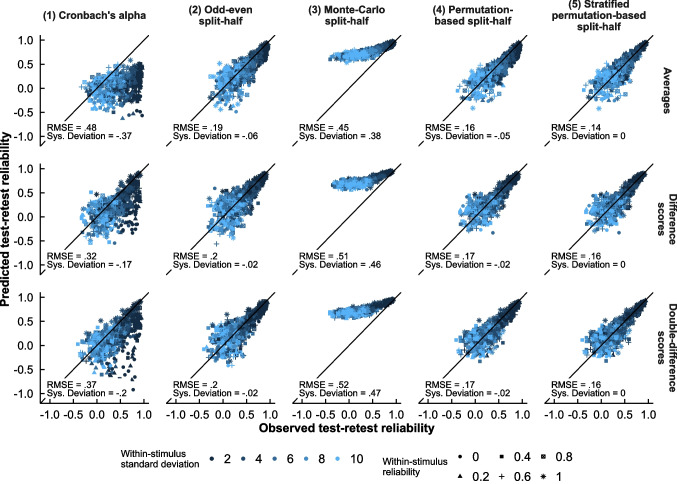
Relationship between the observed test–retest correlation and the predicted test–retest correlation based on the reliability of the individual test and retest datasets. RMSE = root mean square error; sys. deviation = the systematic mean deviation of the predicted value from the observed value, with negative values implying a systematic tendency to underestimate. Points below the diagonal imply that the test–retest correlation was underestimated. The appearance of the points is based on the properties of the simulated dataset: the points are colored based on the *SD* of the RTs belonging to each stimulus, and they are shaped on the basis of the preset (Cronbach’s alpha) reliability of the stimulus-specific bias scores within the two stimulus categories

The results can be viewed in Fig. 5. As expected, Cronbach’s alpha (Panel 1) was highly inaccurate and often strongly underestimated the test–retest correlation. This was especially true for scores based on a simple average, where it showed almost no relationship with the actual reliability. Compared to Cronbach’s alpha, odd–even split-half reliability (Panel 2) predicted the test–retest correlation with less error and less systematic deviation. The unstratified permutation-based split-half reliability (Panel 4) was more accurate and less deviated than both Cronbach’s alpha and odd–even split-half reliability. The stratified variant of the permutation-based split-half (Panel 5) performed best: it was not deviated at all, and slightly more accurate than its unstratified counterpart in all circumstances. The Monte Carlo split-half was highly inaccurate and systematically deviated, such that reliability was always strongly overestimated (Panel 3); it will not be further examined.

#### Factors influencing reliability and its estimation

We also examined how the characteristics of the input datasets affected the observed and predicted test-retest correlations of double-difference scores; these results followed the same pattern for averages and single-difference scores. The true test–retest correlation strongly depended on the variability of RTs (*r* =  − 0.63), a property that Cronbach’s alpha was not as sensitive to (*r* =  − 0.29) as the split-half methods were (odd–even: *r* =  − 0.59; permutation-based: *r* =  − 0.62; stratified permutation-based: *r* =  − 0.64). Rather, Cronbach’s alpha was strongly influenced by the Cronbach’s alpha value of the stimulus-specific bias scores within each stimulus category (*r* = 0.46), a property the split-half methods were barely sensitive to (odd–even: *r* = 0.04; permutation-based: *r* = 0.06; stratified permutation-based: *r* = 0) and which barely influenced the real correlation between the two samples (*r* = 0.01). Hence, the inaccuracy of Cronbach’s alpha is likely due to this sensitivity to components of the data that do not affect the actual reliability of the scores.

#### Summary

We can conclude that reliability cannot be accurately computed in RT tasks by averaging RTs across stimulus repetitions to compute stimulus-specific subscores and computing Cronbach’s alpha with these; we demonstrated that reliability can be more accurately computed with split-half reliability coefficients. Among these, the odd–even split-half was less accurate than its permutation-based counterpart. The stratified permutation-based split-half reliability performed better than its unstratified counterpart, with no detectable systematic deviation and less error. The new Monte Carlo split-half reliability coefficient strongly overestimated reliability and should not be used. We additionally found that the permutation-based split-half reliability coefficients are best computed by aggregating the individual split-half correlations before applying a modified Spearman–Brown formula.

## Study 2: What is the ideal number of splits?

### Introduction and methods

It remains unclear how many splits should be averaged together to compute an accurate permutation-based split-half reliability coefficient—too many splits would take unnecessarily long to compute, while too few splits would produce estimates that are unnecessarily inaccurate and do not replicate. We thus used simulated datasets to find the sufficient number of splits required to obtain a stable permutation-based split-half reliability coefficient under a variety of conditions.

We generated 150 datasets for each permutation of different sample sizes (30, 45, 60, 120), trial counts (64, 128, 256), true underlying reliabilities (0, 0.2, 0.5, 0.8), score types (average, single-difference score, double-difference score), and use of stratification by stimulus (stratified, unstratified), totaling 43,200 datasets. In each dataset we computed 20,000 split-half correlations for scoring methods based on means, medians, and *D* scores (i.e., means or mean-based difference scores divided by the participant’s overall *SD*). From these 20,000 split-half correlations, we randomly sampled values in sets that were enlarged incrementally in steps of 100. The correlations in these sets were averaged, and it was recorded how strongly this average deviated from the grand average of 20,000 split-half correlations. For each combination of algorithm, true reliability, sample size, and trial count, we computed the largest number of averaged-together split-halves at which 95% of the averages deviated less than 0.01 from their equivalent grand average of 20,000 correlations.

### Results and discussion

**Fig. 6:**
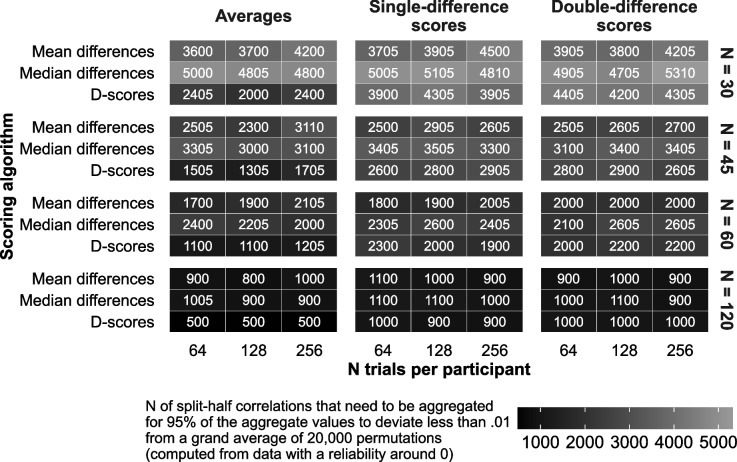
Minimal number of split-half correlations required to obtain an accurate permutation-based split-half reliability estimates given a true reliability of 0. Less splits were required in tasks in which the true reliability was higher than 0

Reliabilities with and without stratification required approximately equal numbers of splits under all circumstances, and were thus collapsed. Required permutation count was dependent on the true reliability of the studied effect, with more reliable effects requiring less splits to estimate. Since this true reliability is not known beforehand, we display the numbers of required splits for effects with a true reliability of 0 in Fig. 6.

The number of required split-half correlations depended on the number of participants, with less splits required when there were more participants in the data; in addition, median double difference scores required more splits than the other algorithms, and *D* scores required less splits than the other algorithms when computing the reliability of simple averages. Importantly, reliabilities were accurate under any examined condition given at least 5,400 splits. This is more than we used in one of our previous studies (Kahveci et al., 2020a). Hence, these findings should aid researchers in planning how many splits to use when computing the permutation-based split-half reliability of their tasks.

## Study 3: The utility of split-half reliability confidence intervals

### Introduction and methods

Since the permutation-based split-half reliability coefficient is an average of thousands of individual random split-half correlations, does this mean that the coefficient is more likely to be inaccurate when its constituent correlations are more dispersed? The importance of this question is highlighted by the fact that a number of R implementations of permutation-based split-half reliability customarily report the 95% confidence interval (95% CI) of the original correlations, even though it remains to be ascertained whether it actually represents a measure of estimate accuracy. This is what we investigated in the current study.

We generated 200 pairs of datasets for each permutation of score for which to compute the reliability (simple averages, single-difference score, double-difference score), true score variability between subjects (none, medium, large), and sample size (30, 60, 120, 240), giving a total of 7,200 datasets. Each participant featured eight target and eight control stimuli that were each ‘displayed’ eight times in the approach condition and eight times in the avoid condition, thus giving 256 trials per participant per dataset. Again, each pair of datasets featured the same set of participants with the same properties. Stratified permutation-based split-half reliabilities were computed using 10,000 splits and with stratification on the basis of stimulus ID. The predicted and observed test–retest correlation were computed in the same manner as in Study 1. We computed the 95% CI of the reliabilities by taking the 2.5% and 97.5% quantiles of the individual split-half correlations and submitting them to the modified Spearman–Brown formula. We then computed the 95% CI of the predicted test–retest correlation by averaging together the 95% CIs of the two reliability values.

### Results and discussion

**Fig. 7:**
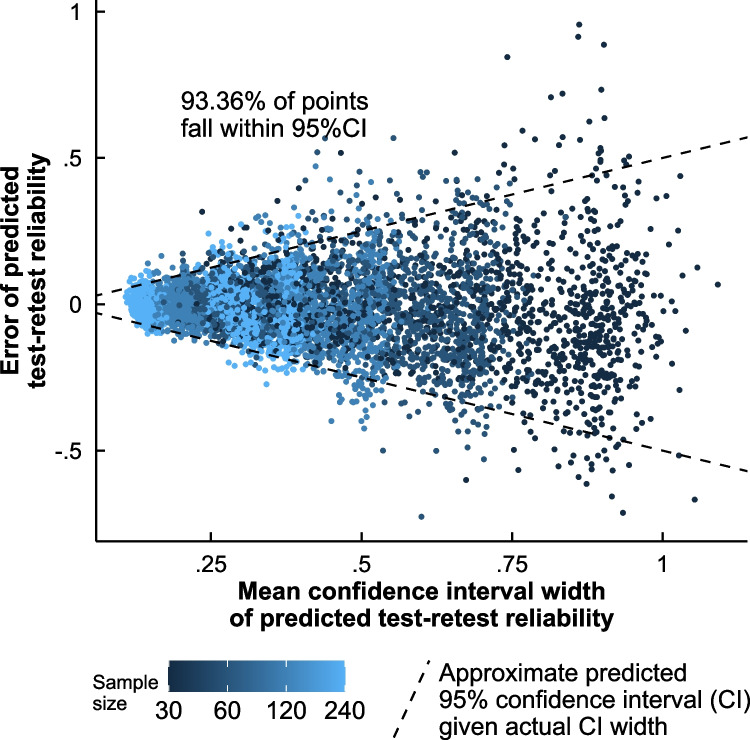
Error of the stratified permutation-based split-half reliability in predicting the test–retest correlation, as a function of the width of the 95% confidence intervals of its predicted test–retest correlations

As depicted in Fig. 7, the range of prediction errors increased with wider permutation-based 95% CIs. However, 6.64% of observed test–retest correlations fell outside the permutation-based 95%CI of the predicted test–retest correlations, suggesting that they may slightly underestimate error. The 95% CIs were too narrow in all types of Spearman–Brown formula (discussed in Study 1), and regardless of the type of score analyzed, be it average or difference score. When the 95% CIs were computed analytically rather than with the actual distribution of split-half correlations, 12.9% of observations fell outside the intervals, suggesting that the permutation-based 95% CI at least performs better.

The 95% CI width was influenced by the same aspects of the data that the actual test–retest correlation prediction error was influenced by. The 95% CIs were wider with smaller sample sizes, *r*(5843) =  − 0.62, *p* < 0.001, and with smaller predicted test–retest correlations, *r*(5843) =  − 0.65, *p* < 0.001. Likewise, the actual prediction error also became larger with smaller sample sizes, *r*(5843) =  − 0.31, *p* < 0.001; and with smaller predicted test–retest correlations, *r*(5843) =  − 0.42, *p* < 0.001. In Table 1, we give an overview of the average absolute prediction error of the test–retest correlation, as a function of sample size and predicted test–retest correlation; the values indicate that accurately estimating a task’s reliability will require larger samples in unreliable tasks than in reliable ones.

**Table 1:** Average absolute error of the predicted test–retest correlation given different sample sizes and predicted test–retest correlation sizes

Sample size	Average absolute error of predicted test–retest correlation given different values of predicted test–retest correlation			
	0 to 0.25	0.25 to 0.5	0.5 to 0.75	0.75 to 1
30	0.19	0.16	0.09	0.08
60	0.17	0.12	0.06	0.04
120	0.14	0.08	0.04	0.05
240	0.09	0.06	0.03	–

## Study 4: Impact on the literature

### Introduction and methods

Since Cronbach’s alpha has often been used to compute reliability in reaction-time tasks, the question arises of how the current findings impact the interpretation of these previous studies. We illustrated the impact of the current findings by reanalyzing a number of studies that used Cronbach’s alpha, using the recommended stratified permutation-based split-half reliability coefficient. We also compared Cronbach’s alpha and split-half reliability values in studies that reported both.

#### Reanalysis

We contacted the authors of 20 RT studies in which the primary single-session reliability coefficient was a covariance-based coefficient like Cronbach’s alpha or McDonald’s omega; among these, we received the data of three studies (Bohne et al., 2021; Kersbergen et al., 2015; Watson et al., 2013), and another study author (Jones et al., 2018) offered to analyze three studies on our behalf given ethical barriers to sharing the original datasets (Field et al., 2007, 2009; Schoenmakers et al., 2008). For these datasets, we or the author computed Cronbach’s alpha, odd–even split-half reliability, as well as permutation-based and stratified permutation-based split-half reliability with 10,000 splits in the manner described previously in this manuscript. If the original authors preprocessed their data when computing scores, but did not do so when computing Cronbach’s alpha, we separately computed the different split-half reliabilities with both pre-processed and raw data.

#### Meta-analysis

Due to the low rate of dataset acquisition, we supplemented our reanalysis with split-half reliability (of any kind) and Cronbach’s alpha values taken from seven RT study manuscripts that report both (Cooper et al., 2011; De Houwer & De Bruycker, 2007; Klonteig et al., 2024; Li et al., 2021; Schmukle, 2005; Staugaard, 2009; Waechter et al., 2014). In cases where odd–even split-half reliabilities were not corrected for test length with the Spearman–Brown formula, this was done by us; additionally, in cases where Cronbach’s alpha was left-bounded at zero, the same left-bound was applied to the split-half reliability for comparability. These values were analyzed together with those obtained from raw datasets as described in the previous paragraph. In cases where multiple variants of the split-half reliability were available, we included in our analysis only the coefficient that came out most accurately in Study 1.

### Results and discussion

**Fig. 8:**
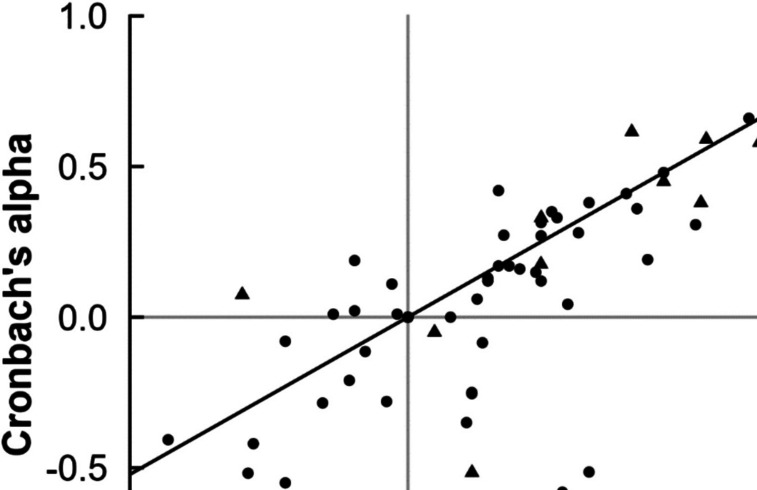
Relationship between Cronbach’s alpha and split-half reliability values across various RT task studies. Each point represents a pair of reliability values for a single measure either reported in the manuscripts included in the meta-analysis or computed from raw data. All values indicated by “Raw data,” including the Cronbach’s alpha values, are computed from the raw data by us or the original study authors using our scripts

We plotted 72 Cronbach’s alpha values from the literature and 17 from the available raw datasets against their equivalent split-half reliability coefficients in Fig. 8. The literature partially replicates our observation from Study 1 that Cronbach’s alpha is often (too) small: split-half reliability was larger in 60% of cases, Cronbach’s alpha was larger in 24% of cases, and the two values were both 0 in 17% of cases.^1^ On average, the split-half reliability coefficient was 0.08 higher than Cronbach’s alpha. We did not, however, observe the extremely deviated Cronbach’s alpha values (> 1 too low) that we observed in Study 1, highlighting that our simulations may have overemphasized a worst-case scenario that rarely occurs in real data.

Besides the aforementioned distortions, our analyses also illustrate how the different methods can produce disparate values when applied to the same data. For example, for a relevant-feature AAT we obtained a Cronbach’s alpha of 0.68 as well as an odd–even *r* = 0.92 and a permutation-based *r* = 0.79 (Kersbergen et al., 2015). The largest contradiction with the original studies was a Cronbach’s α = 0.41 reported in the literature, contrasting with split-half reliabilities around 0.3 higher, with odd–even *r* = 0.73, permutation-based *r* = 0.69, and stratified permutation-based *r* = 0.72 (Watson et al., 2013). Given the findings of Study 1, we can assume that the value of the stratified permutation-based split-half reliability will be closest to the true reliability. We list all computed reliabilities in Table 3 in Appendix A.

## General discussion

### Cronbach’s alpha and split-half reliability compared

In four studies, we compared a number of reliability coefficients using simulations and real data, we determined the optimal number of splits for the permutation-based split-half reliability coefficient, and we confirmed the accuracy of its 95% CIs. In Study 1 we found that excessively low reliability estimates are obtained by the common practice of creating averages or bias scores based on subsets of RTs (e.g., across stimulus repetitions) and computing Cronbach’s alpha from these averaged subsets. In Study 4, we confirmed that Cronbach’s alpha values are also frequently lower than split-half reliability in studies that report both. Our findings thus call into question the reliability values reported by many studies (Brom et al., 2014; Christiansen et al., 2015; Lobbestael et al., 2016; Schiebel et al., 2022; Spanakis et al., 2019; van Ens et al., 2019; Watson et al., 2013), some of which had reliability as their centerpiece (e.g., Ataya et al., 2012). Given our findings, we can conclude that Cronbach’s alpha values for RT tasks are likely to underestimate reliability, especially when researchers did not exclude outliers or errors to compute it (Study 4).

A more accurate impression of task reliability can be gleaned from split-half reliabilities. We found that odd–even split-half reliability was much less negatively biased than Cronbach’s alpha, but it was nonetheless somewhat inaccurate. More accurate reliability estimates were obtained by averaging the correlations of many random splits together to produce a permutation-based split-half, especially when we used stratification to ensure that the splits featured the same number of trials per stimulus within each person. We thus recommend that the stratified permutation-based split-half be used wherever possible. An added benefit of this method is that it provides permutation-based confidence intervals that are more accurate than their analytically derived counterparts (Study 3). We also found that the new Monte Carlo split-half reliability coefficient (Pronk et al., 2022) systematically overestimates reliability and should not be used; fortunately, it appears that this method has not seen use in the literature so far.

### Aspects affecting the accuracy of permutation-based split-half reliability

Our findings across this manuscript give an overview of how the permutation-based split-half reliability can be computed accurately. In Study 3, we confirmed that this coefficient is more accurate when sample sizes are larger, and when the analyzed score is more reliable; unreliable tasks and small sample sizes produced more inaccurate reliability estimates. Larger sample sizes are thus needed to accurately estimate reliability in unreliable tasks; the values provided in Table 1 can aid in estimating how large a sample may be needed. In Study 2 we likewise found that more permutation-based split-half correlations are needed to obtain a precise reliability estimate when true reliability and sample size are lower, with the latter displayed in Fig. 6. Safe recommendations for the number of splits include 1,100 for a sample size of 120; 2,600 for a sample size of 60; 3,500 for a sample size of 45; and 5,400 for a sample size of 30. Fewer splits were required for more reliable tasks. Given these findings, we can conclude that one of our earlier reports on task reliability used too few splits and included too few participants to accurately estimate the very low reliability of one of the reported tasks (1,000 splits; Kahveci et al., 2020b); hence, this is a timely finding.

We additionally found in Study 1 that more accurate permutation-based split-half reliability estimates are obtained by aggregating the raw correlations (including negative ones) and then applying the modified Spearman–Brown formula reported in Eq. 6. We further noted in the Introduction that previous research has shown that simple averages of correlation coefficients are negatively biased and that specialized aggregation methods (Olkin & Pratt, 1958) can be used to avert this (Shieh, 2010). Our full list of recommendations is listed in Table 2.

**Table 2:** Recommendations on the use of different reliability coefficients in RT Tasks

Coefficient	Use-case	Prerequisites
Cronbach’s alpha	Never	• No trials are removed as outliers or due to incorrect responses • An equal number of trials is available for each participant • No trials are averaged together • A lower bound of the reliability is desired rather than the reliability itself
Monte Carlo split-half	Never	
Odd–even or first–second split-half	Not when better methods are available	• The modified Spearman–Brown formula in Eq. 6 should be used
Permutation-based split-half	All cases	• A sufficient number of splits should be computed (a safe number is 5,400) • Split-half correlations should ideally be aggregated using an appropriate method (e.g., OP5 from Shieh, 2010) • The modified Spearman–Brown formula in Eq. 6 should be applied after aggregating the raw correlations • Splits should ideally be stratified by stimulus and other grouping variables whose trial counts are expected to remain the same in a hypothetical replication
Test–retest reliability	Whenever a variable has been measured twice per person without reason to expect its value should change	

### Practical application

Permutation-based split-half reliability can be computed from varying-length long-format data using the function splithalf() from R package splithalf (Parsons, 2021), the function by_split() from R package splithalfr (Pronk, 2023), the function aat_splithalf() from R package AATtools (Kahveci, 2022), and the function rapidsplit() from R package rapidsplithalf (Kahveci, 2024). The packages vary in which recommendations from Table 2 they incorporate: AATtools and rapidsplithalf by default use 6,000 splits and the unbiased correlation aggregation method from Eq. 3 (Olkin & Pratt, 1958; Shieh, 2010). These two packages, as well as splithalf, also use the modified Spearman–Brown correction from Eq. 6. Stratified splitting is further supported by AATtools, rapidsplithalf, and splithalfr, though only the former two packages ensure equal numbers of trials in each split half. The rapidsplithalf package also makes a number of interventions from Table 2 available as functions: The unbiased correlation aggregation method from Eq. 3 is available in the function cormean(), the mirrored Spearman–Brown coefficient from Eq. 6 is available in the function spearmanBrown(), and the balanced stratified splitting algorithm is available in the function generateSplits(). In Appendix A, we include a more detailed discussion of the features and a comparison of the accuracy of splithalf, splithalfr, and rapidsplithalf.

Given a hypothetical AAT dataset, one can obtain the stimulus-stratified permutation-based split-half reliability of a double-difference score of median RTs with formula [(avoid food − approach food) − (avoid objects − approach objects)], by using a call in rapidsplithalf that looks like this:
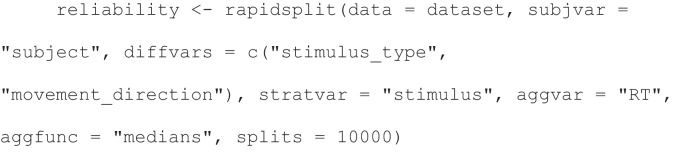


In this R function call, the long-format dataset is contained in dataset as argument to data. Of this dataset, the participant-identifying column is named “subject” as argument to subjvar (subject variable), the RT column is named “RT” as argument to aggvar (aggregation variable), the stimulus-identifying column is named “stimulus” as argument to stratvar (stratification variable), and there are two variables given as argument to diffvars (difference score variables): the stimulus category column is named “stimulus_type”, the movement direction column is named “movement_direction”; the provision of two variables to this argument gives the reliability of a double-difference score as shown in the formula given above, whilst the provision of a single variable would give the reliability of a simple difference score. Further arguments include splits, which here indicates that 10,000 split-half correlations should be aggregated to compute the reliability; and aggfunc, which indicates here that the RTs per condition should be aggregated with medians before the conditions are subtracted from each other. Following computation, the reliability and its confidence intervals can be obtained with the call print(reliability)and a scatterplot can be graphed with plot(reliability)to spot outliers. A full walkthrough of this package is available by calling vignette(“rapidsplithalf”, package = “rapidsplithalf”).

### Limitations and future directions

Even though stratified permutation-based split-half reliability came out as the most accurate and least biased reliability coefficient, its permutation-based 95% confidence intervals were a bit too narrow, as they included 93.36% of all observed test–retest correlations instead of the desired 95%. Hence, the use of these confidence intervals may come with a Type I error above 5%. Analytically computed confidence intervals were unfortunately far narrower, leaving the permutation-based 95% CI as the most accurate confidence interval method available. A further search for an improved confidence interval method fell outside the scope of this manuscript; hence, this task is left to further research.

One further limitation is that the tested reliability coefficients and simulations assume that every observation is independent aside from originating from the same participant. In RT data, this assumption doesnot hold: Consecutive RTs are autocorrelated and influenced by temporal trends caused by learning and fatigue. In such autocorrelated data, there could therefore be a risk that reliability is overestimated and data turns out not to correlate as well as the reliability coefficient might have suggested. Further research^2^ is needed to determine whether autocorrelation actually makes reliability estimates inaccurate, and what methods can nevertheless estimate it accurately. Despite this limitation of the current work, we believe that the conclusions drawn from Study 1 remain valid, since all examined measures can be expected to be affected by autocorrelation to a similar extent.

It further remains unexplored whether preprocessing steps such as outlier exclusion should be applied *after* splitting the data, rather than before—doing so would prevent that information about the presplit data, such as its mean and *SD*, leaks into the split datasets and inflates the reliability (see Yagis et al., 2021, for a discussion of data leakage in cross-validation).

Lastly, a related question is how to *improve* the reliability of cognitive bias measures. We recently evaluated whether specific data preprocessing approaches can improve the reliability of the AAT (Kahveci et al., 2023), but it remains to be investigated whether RT models such as drift diffusion can improve its reliability further: Though diffusion models have been applied to the AAT (e.g., Krypotos et al., 2015), the reliability of their parameters has not yet been evaluated. The reliability of drift diffusion model parameters has recently been explored in the perceptual matching task, however, finding that these parameters may be much less reliable than simple aggregates (Liu et al., 2024). When it comes to task design, it remains mostly unknown which modifications improve reliability, as most meta-analyses focus on effect size, hence signaling the need for meta-analyses to also take reliability into account.

## Open practices statement

The code for generating and analyzing the data for all experiments, as well as the simulated and meta-analysis data, are available online (https://doi.org/10.17605/OSF.IO/YFX2C), and none of the experiments was preregistered. The original datasets analyzed in Study 4 can be obtained from the respective authors.

## Figures and Tables

**Fig. 9:**
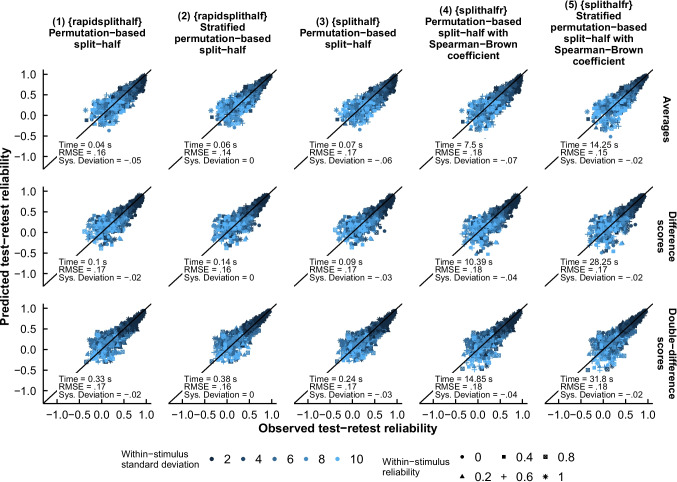
Relationship between the observed and predicted test–retest correlations, with the latter computed using permutation-based split-half reliability from different R packages. Time = average computation time in seconds; RMSE = root mean square error; sys. deviation = the systematic mean deviation of the predicted value from the observed value, with negative values implying a systematic tendency to underestimate. Points below the diagonal imply that the test–retest correlation was underestimated. The appearance of the points is based on the properties of the simulated dataset: the points are colored based on the *SD* of the RTs belonging to each stimulus, and they are shaped on the basis of the preset (Cronbach’s alpha) reliability of the stimulus-specific bias scores within the two stimulus categories. Curly brackets indicate R package names

## Data Availability

All simulated and meta-analysis data that support the findings of this study are openly available in this study's Open Science Foundation repository at 10.17605/OSF.IO/YFX2C. The original datasets analyzed in Study 4 can be obtained from the respective authors.

## Appendix A

### Study 1 data simulation procedure

We aimed to generate data with prespecified covariances between stimulus-specific bias scores and prespecified variances in within-stimulus RTs. First, a covariance matrix was generated representing the covariance between stimulus-specific bias scores for stimuli from two categories, with a prespecified within-category Cronbach’s alpha (α_A_, α_B_), within-category variance (*s*^2^_A_, *s*^2^_B_), within-category stimulus counts (*n*_A_, *n*_B_), and between-category correlation (*r*_AB_). This covariance matrix is displayed in Eq. 7.

7 $${\varvec{S}}=\left(\begin{array}{cccccc}\frac{{s}_{A}^{2}}{{m}_{A}}& \cdots & \frac{{\alpha }_{A}{s}_{A}^{2}}{{m}_{A}({\alpha }_{A}+{n}_{A}-{\alpha }_{A}{n}_{A})}& \frac{{{s}_{A}{s}_{B}r}_{AB}}{{n}_{A}{n}_{B}}& \cdots & \frac{{{s}_{A}{s}_{B}r}_{AB}}{{n}_{A}{n}_{B}}\\ \vdots & \ddots & \vdots & \vdots & \ddots & \vdots \\ \frac{{\alpha }_{A}{s}_{A}^{2}}{{m}_{A}({\alpha }_{A}+{n}_{A}-{\alpha }_{A}{n}_{A})}& \cdots & \frac{{s}_{A}^{2}}{{m}_{A}}& \frac{{{s}_{A}{s}_{B}r}_{AB}}{{n}_{A}{n}_{B}}& \cdots & \frac{{{s}_{A}{s}_{B}r}_{AB}}{{n}_{A}{n}_{B}}\\ \frac{{{s}_{A}{s}_{B}r}_{AB}}{{n}_{A}{n}_{B}}& \cdots & \frac{{{s}_{A}{s}_{B}r}_{AB}}{{n}_{A}{n}_{B}}& \frac{{s}_{B}^{2}}{{m}_{B}}& \cdots & \frac{{\alpha }_{B}{s}_{B}^{2}}{{m}_{B}({\alpha }_{B}+{n}_{B}-{\alpha }_{B}{n}_{B})}\\ \vdots & \ddots & \vdots & \vdots & \ddots & \vdots \\ \frac{{{s}_{A}{s}_{B}r}_{AB}}{{n}_{A}{n}_{B}}& \cdots & \frac{{{s}_{A}{s}_{B}r}_{AB}}{{n}_{A}{n}_{B}}& \frac{{\alpha }_{B}{s}_{B}^{2}}{{m}_{B}({\alpha }_{B}+{n}_{B}-{\alpha }_{B}{n}_{B})}& \cdots & \frac{{s}_{B}^{2}}{{m}_{B}}\end{array}\right)$$

where $${m}_{A}={n}_{A}+{n}_{A}({n}_{A}-1)\frac{{\alpha }_{A}}{{\alpha }_{A}+{n}_{A}-{\alpha }_{A}{n}_{A}}$$  

A dataset of 40 random participants was generated from this covariance matrix, containing stimulus-specific bias scores (*Bias*) for each participant. From these bias scores, we generated random approach and avoidance trials with a preset *SD* (*SDRT*), such that:

$${RT}_{pull, i} \sim ExGauss2\left(\frac{{Bias}_{i}}{2}-\gamma \cdot \tau ,\gamma \cdot \sigma ,\gamma \cdot \tau \right)$$ and $${RT}_{push, i} \sim ExGauss2\left(-\frac{{Bias}_{i}}{2}-\gamma \cdot \tau ,\gamma \cdot \sigma ,\gamma \cdot \tau \right)$$ where $$\gamma =\frac{SDRT}{\sqrt{{\sigma }^{2}+{\tau }^{2}}}$$.

We used this parameterization of the ex-Gaussian distribution, with σ = 1 and τ = 3, to generate right-skewed RT distributions whilst obtaining values with *SD*s equal to *SDRT* and means equal to $\frac{{Bias}_{i}}{2}$ or $-\frac{{Bias}_{i}}{2}$.

Testing confirmed that, when RT sampling was made to conform to the exact covariance matrix and means, the original stimulus-specific bias scores could be recovered from the approach and avoidance trials, the original covariance matrix could be recovered from these bias scores, and the reliability according to Cronbach’s alpha was identical when computed from the input parameters and from the data. In the actual study, we allowed the sampled bias scores and RTs to deviate from the prespecified means and *SD*s in accordance with random sampling.

We used Eq. 8, simplified version of Eq. 7, to generate single-difference scores and simple means with a specific reliability:

8 $${\varvec{S}}=\left(\begin{array}{ccc}\frac{{s}^{2}}{m}& \cdots & \frac{\alpha \cdot {s}^{2}}{m(\alpha +n-\alpha \cdot n)}\\ \vdots & \ddots & \vdots \\ \frac{\alpha \cdot {s}^{2}}{m(\alpha +n-\alpha \cdot n)}& \cdots & \frac{{s}^{2}}{m}\end{array}\right)$$

where $$m=n+n(n-1)\frac{\alpha }{\alpha +n-\alpha \cdot n}$$.

### Data simulation procedure in Studies 2 and 3

A different data simulation procedure was employed for Studies 2 and 3 since different properties were desired from the simulator. Data was generated in two steps: First, we generated participants, each with unique properties; after this, we generated trial-level data for each participant adhering to these properties. The following properties were generated per participant:

- Base RT (samplewide value + normally distributed jitter)
- *SD* of noise parameter (samplewide value + gamma-distributed jitter with shape = 3, rate = 1, and mean = 0)
- Stimulus-independent approach-avoidance RT difference (samplewide value + normally distributed jitter)
- Movement-independent stimulus category RT difference (samplewide value + normally distributed jitter)
- Movement-by-stimulus interaction effect (samplewide value + normally distributed jitter)

It was assumed that each participant encounters two stimulus categories (coded 1 and − 1), and that all stimuli are approached and avoided (coded 1 and − 1); furthermore, it was assumed that trials are divided into two blocks. When generating individual trials, the following parameters were also set:

- Stimulus count per stimulus category
- Repetitions per stimulus within movement condition
- Autocorrelation of noise parameter within each block

The default values used for each of the above variables were taken from (Lender et al., 2018). RTs were generated according to the following formula:

$$RT=MeanRT+\frac{1}{2}StimCategory\cdot StimulusEffect+\frac{1}{2}MovementDirection\cdot MovementEffect+\frac{1}{4}StimCategory\cdot MovementDirection\cdot BiasEffect+Error$$

The error variable was generated with an Ex-Gaussian distribution with parameters set such that $\upbeta =\frac{9}{10}SD$, $\upsigma =\sqrt{{SD}^{2}-{\beta }^{2}}$, and $\upmu =Mean-\beta$ to generate right-skewed distributions.

### Reliabilities computed in Study 4

**Table 3:** Overview of the split-half reliabilities computed in Study 4 from preexisting datasets, alongside the original reliability values reported in the articles

Study	Outliers and errors	Variable	Original	Odd–even	Permutation-based	Stratified Permutation-based
Watson et al., 2013	Kept	Smoking ApB	α = 0.41	0.73	0.69	0.72
	Kept	Control ApB	α = 0.46	0.7	0.69	0.71
	Removed	Smoking ApB		0.84	0.65	0.68
	Removed	Control ApB		0.8	0.7	0.72
Kersbergen et al., 2015	Kept	Relevant AAT	α = 0.70	0.92	0.79	0.78
	Kept	Irrelevant AAT	α = − 0.77	− 0.55	− 0.51	− 0.52
	Kept	Relevant SRC	α = 0.58	0.55	0.59	0.6
	Kept	Irrelevant SRC	α = − 0.05	− 0.04	− 0.13	− 0.15
	Removed	Relevant AAT		0.81	0.75	0.76
	Removed	Irrelevant AAT		− 0.41	− 0.22	− 0.24
	Removed	Relevant SRC		0.62	0.65	0.66
	Removed	Irrelevant SRC		0.13	0.07	0.05
Bohne et al., 2021*	Kept	Sad MDD AtB	ω = 0.74	0.69	0.7	0.68
	Kept	Neutral MDD AtB	ω = 0.70	0.7	0.73	0.74
	Kept	Happy MDD AtB	ω = 0.57	0.66	0.63	0.62
	Kept	Sad HC AtB	ω = 0.58	0.54	0.46	0.49
	Kept	Neutral HC AtB	ω = 0.71	0.68	0.53	0.57
	Kept	Happy HC AtB	ω = 0.48	0.55	0.49	0.5
	Removed	Sad MDD		0.8	0.72	0.7
	Removed	Neutral MDD		0.85	0.75	0.76
	Removed	Happy MDD		0.46	0.57	0.56
	Removed	Sad HC		0.68	0.52	0.55
	Removed	Neutral HC		0.84	0.73	0.73
	Removed	Happy HC		0.41	0.46	0.48
Field et al., 2007	Removed (2 *SD*)	Baseline	ω = 0.55	0	− 0.28	− 0.31
Schoenmakers et al., 2008	Removed (2 *SD*)	Alcohol	ω = 0.57	0.33	0.48	0.42
	Removed (2 *SD*)	Placebo	ω = 0.57	0.25	0.12	0.12
Field et al., 2009	Removed (2 *SD*)	50 ms	ω = 0.17	− 0.01	0.23	0.25
	Removed (2 *SD*)	500 ms	ω = 0.46	0.02	0.24	0.25

ApB = approach bias; AtB = attentional bias; AAT = approach–avoidance task; SRC = stimulus–response compatibility task; MDD = major depressive disorder; HC = healthy controls; * = we were unable to properly replicate the McDonald’s omega values reported in the manuscript, and hence our split-half values may differ from those in the original paper not only because of differences between the methods but also other factors

### Comparison of different R packages for computing permutation-based split-half reliability

We used the same methodology as in Study 1 to compare the accuracies of the permutation-based split-half reliability coefficients from R packages splithalf (Version 0.8.2; Parsons, 2021), splithalfr (Version 2.2.2; Pronk, 2023), and rapidsplithalf (Version 0.3; Kahveci, 2024)*.* AATtools (Kahveci, 2022) is not included in this comparison since its implementation is mathematically identical to that of rapidsplithalf. Whilst splithalf and rapidsplithalf produced a split-half reliability value from a single function call, splithalfr required more input from the user, so we used the procedures recommended by the package authors, including the use of a simple mean to average the reliabilities. We also compared the three different split-half reliability coefficients offered by this package, including the original Spearman–Brown coefficient, the Angoff–Feldt coefficient, and the Flanagan–Rulon coefficient. All splits in splithalfr were stratified by condition, as occurs by default in rapidsplithalf; additionally, we computed a stratified permutation-based split-half from both the rapidsplithalf and splithalfr packages. We computed 100 splits for each coefficient (rather than the recommended 5,400) since the splithalfr package was significantly slower than the other two packages and more splits would not have been feasible to compute in this study. All analyses were run single-threaded and the computation time was recorded.

The results are displayed in Fig. 9; the Angoff–Feldt and Flanagan–Rulon coefficients from splithalfr performed practically identical to the Spearman–Brown coefficient and are thus omitted. Among the unstratified split-half methods, rapidsplithalf and splithalf did not strongly differ in error, but rapidsplithalf was slightly less systematically deviated; splithalfr showed more error and more systematic deviation. The stratified split-half coefficients of rapidsplithalf and splithalfr were more accurate and less systematically deviated than their unstratified counterparts, but, again, the rapidsplithalf coefficient had less systematic deviation and error. In terms of computation time, the splithalfr package stood out as extremely slow compared with the other two packages: computing 100 stratified split-halves took over half a minute on average, compared with subsecond computation times for the other two packages.

Though rapidsplithalf came out most accurate and least deviated, it must be emphasized these values only apply to the tested versions of these R packages and are subject to change with new releases. In addition, it must be noted that splithalfr, unlike the other two packages, supports computing the reliability of virtually any scoring method, and is thus indicated when the other two packages do not support the desired scoring method. Further, a feature of the splithalf package lacked by the other two packages is its support for multiverse analysis (i.e., the exploration of how reliability is affected by different data preprocessing methods).

## Footnotes

1 The reported counts are based on reliabilities taken from the literature, and on computed reliabilities from data without outliers and errors; however, the pattern was the same when we instead used the computed split-half reliabilities based on data with outliers and errors included: split-half reliability was larger in 60% of cases, Cronbach’s alpha was larger in 24% of cases, and both values were zero in 17% of cases.

2 We ran an exploratory simulation of our own, which produced highly implausible findings. It is available in the osf.io repository of this project.

## References

[CR1] Ataya, A. F., Adams, S., Mullings, E., Cooper, R. M., Attwood, A. S., & Munafò, M. R. (2012). Internal reliability of measures of substance-related cognitive bias. *Drug and Alcohol Dependence,**121*(1), 148–151. 10.1016/j.drugalcdep.2011.08.023 21955365 10.1016/j.drugalcdep.2011.08.023

[CR2] Atwood, S., & Axt, J. R. (2021). Assessing implicit attitudes about androgyny. *Journal of Experimental Social Psychology*, *96*, Article 104162. 10.1016/j.jesp.2021.104162

[CR3] Bohne, A., Nordahl, D., Lindahl, Å. A. W., Ulvenes, P., Wang, C. E. A., & Pfuhl, G. (2021). Emotional infant face processing in women with major depression and expecting parents with depressive symptoms. *Frontiers in Psychology*, *12*. 10.3389/fpsyg.2021.657269 10.3389/fpsyg.2021.657269 PMC8283203 34276481

[CR4] Brom, M., Laan, E., Everaerd, W., Spinhoven, P., & Both, S. (2014). Extinction and renewal of conditioned sexual responses. *PLOS ONE*, *9*(8), Article e105955. 10.1371/journal.pone.0105955 10.1371/journal.pone.0105955 PMC4149496 25170909

[CR5] Chapman, A., Devue, C., & Grimshaw, G. M. (2019). Fleeting reliability in the dot-probe task. *Psychological Research Psychologische Forschung,**83*(2), 308–320. 10.1007/s00426-017-0947-6 29159699 10.1007/s00426-017-0947-6

[CR6] Christiansen, P., Mansfield, R., Duckworth, J., Field, M., & Jones, A. (2015). Internal reliability of the alcohol-related visual probe task is increased by utilising personalised stimuli and eye-tracking. *Drug and Alcohol Dependence,**155*, 170–174. 10.1016/j.drugalcdep.2015.07.672 26239377 10.1016/j.drugalcdep.2015.07.672

[CR7] Cooper, R. M., Bailey, J. E., Diaper, A., Stirland, R., Renton, L. E., Benton, C. P., . . . Munafò, M. R. (2011). Effects of 7.5% CO2 inhalation on allocation of spatial attention to facial cues of emotional expression. *Cognition and Emotion*, *25*(4), 626–638. 10.1080/02699931.2010.508887 10.1080/02699931.2010.508887 21547765

[CR8] Cousijn, J., Goudriaan, A. E., & Wiers, R. W. (2011). Reaching out towards cannabis: Approach-bias in heavy cannabis users predicts changes in cannabis use. *Addiction,**106*(9), 1667–1674. 10.1111/j.1360-0443.2011.03475.x 21518067 10.1111/j.1360-0443.2011.03475.x PMC3178782

[CR9] Cousijn, J., Luijten, M., & Wiers, R. W. (2014). Mechanisms underlying alcohol-approach action tendencies: The role of emotional primes and drinking motives. *Frontiers in Psychiatry*, *5*. 10.3389/fpsyt.2014.00044 10.3389/fpsyt.2014.00044 PMC4018525 24834057

[CR10] Cousijn, J., Snoek, R. W. M., & Wiers, R. W. (2013). Cannabis intoxication inhibits avoidance action tendencies: A field study in the Amsterdam coffee shops. *Psychopharmacology (Berl),**229*(1), 167–176. 10.1007/s00213-013-3097-6 23595593 10.1007/s00213-013-3097-6

[CR11] Cousijn, J., van Benthem, P., van der Schee, E., & Spijkerman, R. (2015). Motivational and control mechanisms underlying adolescent cannabis use disorders: A prospective study. *Developmental Cognitive Neuroscience,**16*, 36–45. 10.1016/j.dcn.2015.04.001 25922296 10.1016/j.dcn.2015.04.001 PMC6989823

[CR12] Cummins, J., Hussey, I., & Spruyt, A. (2022). The role of attitude features in the reliability of IAT scores. *Journal of Experimental Social Psychology*, *101*, Article 104330. 10.1016/j.jesp.2022.104330

[CR13] De Houwer, J. (2006). What are implicit measures and why are we using them? In R. W. Wiers & A. W. Stacy (Eds.), *Handbook of implicit cognition and addiction* (pp. 11–28). SAGE Publications. 10.4135/9781412976237.n2

[CR14] De Houwer, J., & De Bruycker, E. (2007). The identification-EAST as a valid measure of implicit attitudes toward alcohol-related stimuli. *Journal of Behavior Therapy and Experimental Psychiatry,**38*(2), 133–143. 10.1016/j.jbtep.2006.10.004 17109815 10.1016/j.jbtep.2006.10.004

[CR15] De Houwer, J., Teige-Mocigemba, S., Spruyt, A., & Moors, A. (2009). Implicit measures: A normative analysis and review. *Psychological Bulletin,**135*(3), 347–368. 10.1037/a0014211 19379018 10.1037/a0014211

[CR16] Effting, M., Salemink, E., Verschuere, B., & Beckers, T. (2016). Implicit and explicit measures of spider fear and avoidance behavior: Examination of the moderating role of working memory capacity. *Journal of Behavior Therapy and Experimental Psychiatry,**50*, 269–276. 10.1016/j.jbtep.2015.10.003 26497446 10.1016/j.jbtep.2015.10.003

[CR17] Field, M., Duka, T., Eastwood, B., Child, R., Santarcangelo, M., & Gayton, M. (2007). Experimental manipulation of attentional biases in heavy drinkers: Do the effects generalise? *Psychopharmacology (Berl),**192*(4), 593–608. 10.1007/s00213-007-0760-9 17361393 10.1007/s00213-007-0760-9

[CR18] Field, M., Duka, T., Tyler, E., & Schoenmakers, T. (2009). Attentional bias modification in tobacco smokers. *Nicotine & Tobacco Research,**11*(7), 812–822. 10.1093/ntr/ntp067 19474181 10.1093/ntr/ntp067

[CR19] Heuer, K., Rinck, M., & Becker, E. S. (2007). Avoidance of emotional facial expressions in social anxiety: The approach–avoidance task. *Behaviour Research and Therapy,**45*(12), 2990–3001. 10.1016/j.brat.2007.08.010 17889827 10.1016/j.brat.2007.08.010

[CR20] Jones, A., Christiansen, P., & Field, M. (2018). Failed attempts to improve the reliability of the alcohol visual probe task following empirical recommendations. *Psychology of Addictive Behaviors,**32*(8), 922–932. 10.1037/adb0000414 30475013 10.1037/adb0000414 PMC6296781

[CR21] Kahveci, S. (2022). *AATtools: Reliability and scoring routines for the approach–avoidance task* (Version 0.0.2) [Computer software]. 10.32614/CRAN.package.AATtools

[CR22] Kahveci, S. (2024). *rapidsplithalf: A fast split-half reliability algorithm* (Version 0.3) [Computer software]. 10.32614/CRAN.package.rapidsplithalf

[CR23] Kahveci, S., Meule, A., Lender, A., & Blechert, J. (2020). Food approach bias is moderated by the desire to eat specific foods. *Appetite*, *154*, Article 104758. 10.1016/j.appet.2020.104758 10.1016/j.appet.2020.104758 32535212

[CR24] Kahveci, S., Rinck, M., van Alebeek, H., & Blechert, J. (2023). How pre-processing decisions affect the reliability and validity of the approach–avoidance task: Evidence from simulations and multiverse analyses with six datasets. *Behavior Research Methods*10.3758/s13428-023-02109-1 10.3758/s13428-023-02109-1 PMC10990989 37221345

[CR25] Kahveci, S., van Bockstaele, B., Blechert, J., & Wiers, R. W. (2020). Pulling for pleasure? Erotic approach-bias associated with porn use, not problems. *Learning and Motivation*, *72*, Article 101656. 10.1016/j.lmot.2020.101656

[CR26] Kersbergen, I., Woud, M. L., & Field, M. (2015). The validity of different measures of automatic alcohol action tendencies. *Psychology of Addictive Behaviors,**29*(1), 225–230. 10.1037/adb0000009 25134039 10.1037/adb0000009

[CR27] Klein, A. M., Becker, E. S., & Rinck, M. (2011). Approach and avoidance tendencies in spider fearful children: The approach-avoidance task. *Journal of Child and Family Studies,**20*(2), 224–231. 10.1007/s10826-010-9402-7 21475709 10.1007/s10826-010-9402-7 PMC3048304

[CR28] Klonteig, S., Roalsø, E. S., Jonassen, R., Hilland, E., Moberget, T., Mirtaheri, P., & Kraft, B. (2024). *Measuring attentional bias using the dot-probe task in young women: Psychometric properties and feasibility of response-based computations, dwell time, and the N2pc component*. ResearchSquare. 10.21203/rs.3.rs-4642037/v1 10.1016/j.jbtep.2025.102036 40245588

[CR29] Kong, G., Larsen, H., Cavallo, D. A., Becker, D., Cousijn, J., Salemink, E., . . . Krishnan-Sarin, S. (2015). Re-training automatic action tendencies to approach cigarettes among adolescent smokers: A pilot study. *The American Journal of Drug and Alcohol Abuse*, *41*(5), 425–432. 10.3109/00952990.2015.1049492 10.3109/00952990.2015.1049492 PMC4561007 26186485

[CR30] Krypotos, A.-M., Beckers, T., Kindt, M., & Wagenmakers, E.-J. (2015). A Bayesian hierarchical diffusion model decomposition of performance in approach–avoidance tasks. *Cognition and Emotion,**29*(8), 1424–1444. 10.1080/02699931.2014.985635 25491372 10.1080/02699931.2014.985635 PMC4673543

[CR31] Lender, A., Meule, A., Rinck, M., Brockmeyer, T., & Blechert, J. (2018). Measurement of food-related approach–avoidance biases: Larger biases when food stimuli are task relevant. *Appetite,**125*, 42–47. 10.1016/j.appet.2018.01.032 29407526 10.1016/j.appet.2018.01.032

[CR32] Li, M.-H., Li, P.-W., & Rao, L.-L. (2021). Self–other moral bias: Evidence from implicit measures and the Word-Embedding Association Test. *Personality and Individual Differences*, *183*, Article 111107. 10.1016/j.paid.2021.111107

[CR33] Liu, Z., Hu, M., Zheng, Y.-R., Sui, J., & Chuan-Peng, H. (2024). A multiverse assessment of the reliability of the perceptual matching task as a measurement of the self-prioritization effect. *PsyArXiv Preprints.*10.31234/osf.io/g6uap 10.3758/s13428-024-02538-6 39747721

[CR34] Lobbestael, J., Cousijn, J., Brugman, S., & Wiers, R. W. (2016). Approach and avoidance towards aggressive stimuli and its relation to reactive and proactive aggression. *Psychiatry Research,**240*, 196–201. 10.1016/j.psychres.2016.04.038 27111213 10.1016/j.psychres.2016.04.038

[CR35] Lord, F. M. (1963). Elementary models for measuring change. In C. W. Harris (Ed.), *Problems in measuring change* (pp. 21–38). University of Wisconsin Press.

[CR36] Manchery, L., Yarmush, D. E., Luehring-Jones, P., & Erblich, J. (2017). Attentional bias to alcohol stimuli predicts elevated cue-induced craving in young adult social drinkers. *Addictive Behaviors,**70*, 14–17. 10.1016/j.addbeh.2017.01.035 28161617 10.1016/j.addbeh.2017.01.035

[CR37] Najmi, S., Kuckertz, J. M., & Amir, N. (2010). Automatic avoidance tendencies in individuals with contamination-related obsessive-compulsive symptoms. *Behaviour Research and Therapy,**48*(10), 1058–1062. 10.1016/j.brat.2010.06.007 20650448 10.1016/j.brat.2010.06.007 PMC2930101

[CR38] Olkin, I., & Pratt, J. W. (1958). Unbiased estimation of certain correlation coefficients. *The Annals of Mathematical Statistics,**29*(1), 201–211. 10.1214/aoms/1177706717

[CR39] Parsons, S. (2021). splithalf: Robust estimates of split half reliability. *Journal of Open Source Software*, *6*(60), Article 3041. 10.21105/joss.03041

[CR40] Parsons, S., Kruijt, A.-W., & Fox, E. (2019). Psychological Science Needs a Standard Practice of Reporting the Reliability of Cognitive-Behavioral Measurements. *Advances in Methods and Practices in Psychological Science,**2*(4), 378–395. 10.1177/2515245919879695

[CR41] Pronk, T. (2023). *splithalfr: Estimate split-half reliabilities* (Version 2.2.2) [Computer software]. 10.32614/CRAN.package.splithalfr

[CR42] Pronk, T., Molenaar, D., Wiers, R. W., & Murre, J. (2022). Methods to split cognitive task data for estimating split-half reliability: A comprehensive review and systematic assessment. *Psychonomic Bulletin & Review,**29*(1), 44–54. 10.3758/s13423-021-01948-3 34100223 10.3758/s13423-021-01948-3 PMC8858277

[CR43] Reinecke, A., Becker, E. S., & Rinck, M. (2010). Three indirect tasks assessing implicit threat associations and behavioral response tendencies: Test–retest reliability and validity. *Zeitschrift Für Psychologie/journal of Psychology,**218*(1), 4–11. 10.1027/0044-3409/a000002

[CR44] Rinck, M., & Becker, E. S. (2007). Approach and avoidance in fear of spiders. *Journal of Behavior Therapy and Experimental Psychiatry,**38*(2), 105–120. 10.1016/j.jbtep.2006.10.001 17126289 10.1016/j.jbtep.2006.10.001

[CR45] Rinck, M., Dapprich, A., Lender, A., Kahveci, S., & Blechert, J. (2021). Grab it or not? Measuring avoidance of spiders with touchscreen-based hand movements. *Journal of Behavior Therapy and Experimental Psychiatry*, *73*, Article 101670. 10.1016/j.jbtep.2021.101670 10.1016/j.jbtep.2021.101670 34157656

[CR46] Schiebel, T., Gallinat, J., & Kühn, S. (2022). Testing the biophilia theory: Automatic approach tendencies towards nature. *Journal of Environmental Psychology*, *79*, Article 101725. 10.1016/j.jenvp.2021.101725

[CR47] Schmukle, S. C. (2005). Unreliability of the dot probe task. *European Journal of Personality,**19*(7), 595–605. 10.1002/per.554

[CR48] Schoenmakers, T., Wiers, R. W., & Field, M. (2008). Effects of a low dose of alcohol on cognitive biases and craving in heavy drinkers. *Psychopharmacology (Berl),**197*(1), 169–178. 10.1007/s00213-007-1023-5 18066535 10.1007/s00213-007-1023-5 PMC2493055

[CR49] Shieh, G. (2010). Estimation of the simple correlation coefficient. *Behavior Research Methods,**42*(4), 906–917. 10.3758/BRM.42.4.906 21139158 10.3758/BRM.42.4.906

[CR50] Sijtsma, K. (2009). On the use, the misuse, and the very limited usefulness of Cronbach’s alpha. *Psychometrika,**74*(1), 107–120. 10.1007/s11336-008-9101-0 20037639 10.1007/s11336-008-9101-0 PMC2792363

[CR51] Silver, N. C., & Dunlap, W. P. (1987). Averaging correlation coefficients: Should Fisher’s *z* transformation be used? *Journal of Applied Psychology,**72*(1), 146–148. 10.1037/0021-9010.72.1.146

[CR52] Sklenarik, S. M., Potenza, M. N., & Astur, R. S. (2024). Avoidance biases for vaping stimuli among college students with electronic-cigarette use. *Addictive Behaviors*, *151*, Article 107934. 10.1016/j.addbeh.2023.107934 10.1016/j.addbeh.2023.107934 38101120

[CR53] Spanakis, P., Jones, A., Field, M., & Christiansen, P. (2019). A Stroop in the hand is worth two on the laptop: Superior reliability of a smartphone based alcohol Stroop in the real world. *Substance Use & Misuse,**54*(4), 692–698. 10.1080/10826084.2018.1536716 30572780 10.1080/10826084.2018.1536716

[CR54] Spearman, C. (1904). The proof and measurement of association between two things. *The American Journal of Psychology,**15*(1), 72–101. 10.2307/1412159 3322052

[CR55] Staugaard, S. R. (2009). Reliability of two versions of the dot-probe task using photographic faces. *Psychology Science,**51*(3), 339–350.

[CR56] Struijs, S. Y., Lamers, F., Vroling, M. S., Roelofs, K., Spinhoven, P., & Penninx, B. W. J. H. (2017). Approach and avoidance tendencies in depression and anxiety disorders. *Psychiatry Research,**256*, 475–481. 10.1016/j.psychres.2017.07.010 28715782 10.1016/j.psychres.2017.07.010

[CR57] Szasz, P. L., Szentagotai, A., & Hofmann, S. G. (2012). Effects of emotion regulation strategies on smoking craving, attentional bias, and task persistence. *Behaviour Research and Therapy,**50*(5), 333–340. 10.1016/j.brat.2012.02.010 22459732 10.1016/j.brat.2012.02.010

[CR58] Tabachnick, B. G., Fidell, L. S., & Ullman, J. B. (2019). *Using multivariate statistics* (7th ed.). Pearson.

[CR59] Tabatabaei, F., & Beldona, S. (2024). Are eco-friendly hotels inconvenient? An Implicit Association Test. *Journal of Hospitality and Tourism Management,**58*, 197–208. 10.1016/j.jhtm.2024.01.001

[CR60] Tian, Q., & Smith, J. C. (2011). Attentional bias to emotional stimuli is altered during moderate- but not high-intensity exercise. *Emotion,**11*(6), 1415–1424. 10.1037/a0023568 21707164 10.1037/a0023568

[CR61] Van Bockstaele, B., Lamens, L., Salemink, E., Wiers, R. W., Bögels, S. M., & Nikolaou, K. (2020). Reliability and validity of measures of attentional bias towards threat in unselected student samples: Seek, but will you find? *Cognition and Emotion,**34*(2), 217–228. 10.1080/02699931.2019.1609423 31044648 10.1080/02699931.2019.1609423

[CR62] Van Duijvenbode, N., Didden, R., Korzilius, H. P. L. M., & Engels, R. C. M. E. (2017). Attentional bias in problematic drinkers with and without mild to borderline intellectual disability. *Journal of Intellectual Disability Research,**61*(3), 255–265. 10.1111/jir.12335 27585827 10.1111/jir.12335

[CR63] van Ens, W., Schmidt, U., Campbell, I. C., Roefs, A., & Werthmann, J. (2019). Test–retest reliability of attention bias for food: Robust eye-tracking and reaction time indices. *Appetite,**136*, 86–92. 10.1016/j.appet.2019.01.020 30682381 10.1016/j.appet.2019.01.020

[CR64] Waechter, S., Nelson, A. L., Wright, C., Hyatt, A., & Oakman, J. (2014). Measuring attentional bias to threat: Reliability of dot probe and eye movement indices. *Cognitive Therapy and Research,**38*(3), 313–333. 10.1007/s10608-013-9588-2

[CR65] Watson, P., de Wit, S., Cousijn, J., Hommel, B., & Wiers, R. W. (2013). Motivational mechanisms underlying the approach bias to cigarettes. *Journal of Experimental Psychopathology,**4*(3), 250–262. 10.5127/jep.030512

[CR66] Williams, B. J., & Kaufmann, L. M. (2012). Reliability of the go/no go association task. *Journal of Experimental Social Psychology,**48*(4), 879–891. 10.1016/j.jesp.2012.03.001

[CR67] Yagis, E., Atnafu, S. W., García Seco de Herrera, A., Marzi, C., Scheda, R., Giannelli, M., . . . Diciotti, S. (2021). Effect of data leakage in brain MRI classification using 2D convolutional neural networks. *Scientific Reports*, *11*(1), Article 1. 10.1038/s41598-021-01681-w 10.1038/s41598-021-01681-w PMC8604922 34799630

